# The Evolutionary Landscape of tRNA Modifications in Archaea: Insights from High-Throughput Sequencing

**DOI:** 10.1101/2025.05.02.651894

**Published:** 2025-05-05

**Authors:** Jesse S. Leavitt, Henry Moore, Thomas J. Santangelo, Todd M. Lowe

**Affiliations:** 1 Department of Biomolecular Engineering, Baskin School of Engineering, University of California Santa Cruz, Santa Cruz, CA 95064, USA.; 2 Department of Biochemistry and Molecular Biology, Colorado State University, Fort Collins, CO, 80523, USA.

**Keywords:** Archaea, reverse-transcription, misincorporation, tRNA modification, enzyme-substrate coevolution

## Abstract

Transfer RNA (tRNA) modifications play essential roles in structure, stability and decoding accuracy, yet the evolutionary dynamics and diversity of these modifications remain incompletely understood. Recent advances in high-throughput sequencing, including Ordered Two-Template Relay sequencing (OTTR-seq), now facilitate systematic, transcriptome-wide detection of tRNA modifications at single-base resolution. Here we employed OTTR-seq to comprehensively profile tRNA modifications across nine archaeal species spanning diverse ecological niches, including thermophiles, methanogens, acidophiles, and halophiles. We identified coordinated and mutually exclusive methylation patterns at acceptor stem position 6 and 67 in hyperthermophiles, as well as unexpected clade-specific co-modifications at core positions 10 and 26, demonstrating tolerance of canonical anti-determinants in Thermoprotei. Comparative genomic analyses revealed clear associations between modifications and evolutionary changes in enzyme domain architectures, including divergence in homologs of Trm14, Trm10, Trm11, and Trm1. We also expanded upon previously characterized identity elements in the D-stem, such as the conserved G10oU25 pair critical for Trm11 specificity, providing additional insight into determinants that likely govern enzyme-substrate interactions. Together, these findings offer valuable insights into archaeal tRNA modification biology and evolutionary dynamics, and provide a foundation for guiding targeted biochemical experiments and mechanistic studies.

## Introduction

Transfer RNAs (tRNAs) are central to translating genetic information into functional proteins in all living systems. Post-transcriptional modifications heavily influence tRNA structure and function, modulating the rate of maturation, molecular stability, and the specificity of interactions with aminoacyl-tRNA synthetases, mRNA codons, and a multitude of other cellular components (Grosjean, 2010; Grosjean et al., 2008; Hernandez-Alias et al., 2022; Schultz & Kothe, 2024; Sokołowski et al., 2018; Yared et al., 2024). These modifications are differentially conserved at distinct positions to impart specific properties (Höfer & Jäschke, 2018; Ontiveros et al., 2019). In Archaea, the complete patterns of tRNA modifications are known for only two archaeal species due to the difficulty and low-throughput nature of traditional biochemical characterization methods. From the comprehensive mapping in *Haloferax volcanii* and *Methanocaldococcus jannaschii* (Gupta, 1984; Yu et al., 2019), along with partial mapping in *Methanococcus maripaludis*, *Pyrococcus furiosus*, and *Sulfolobus acidocaldarius* (Wolff et al., 2023), it is clear that tRNA modifications contribute to adaptation in diverse extreme environments, such as high temperatures or low pH, where these organisms reside (Fluke et al., 2024; Hori et al., 2018; Kowalak et al., 1994; Li et al., 2019; Lorenz et al., 2017; Ohira & Suzuki, 2024; Sas-Chen et al., 2020; Shigi, 2018; Tsai et al., 2025).

The archaeal species in this study span diverse ecological environments: *P. furiosus* thrives at temperatures approaching 100°C in deep-sea hydrothermal vents (Schicho et al., 1993), *S. acidocaldarius* is adapted to acid hot springs with pH values near 2 (Reno et al., 2009), and *H. volcanii* grows in hypersaline environments with salt concentrations exceeding 3M NaCl (Gupta, 1984). Even within methanogens like *M. maripaludis* and *M. jannaschii*, adaptation to anoxic and high-pressure marine environments is evident (Whitman et al., 1987). The chemical stability and structural plasticity conferred by tRNA modifications likely play roles in enabling translation to proceed under these diverse and often extreme physicochemical conditions.

High-throughput tRNA sequencing has become a powerful tool for quantifying tRNA abundance and expression dynamics, complementing data from traditional RNA-seq approaches that often underrepresent structured or modified RNAs. In addition to profiling tRNA expression, specialized tRNA-seq methods now enable the detection of chemical modifications by leveraging the tendency of certain base modifications to disrupt Watson-Crick (W-C) base pairing during reverse transcription (RT), which can be captured using various methods (Behrens et al., 2021; Cozen et al., 2015; Erber et al., 2020; Gustafsson et al., 2022; Kimura et al., 2020; Nakano et al., 2025; Upton et al., 2021; Zheng et al., 2015). While mass spectrometry remains the gold standard for direct chemical identification of modified nucleosides, RT-based sequencing offers a complementary high-throughput, nucleotide-resolution approach.

One of the newest RT-based tRNA seq methods, Ordered Two Template Relay sequencing (OTTR-seq), enhances detection of modification-sensitive misincorporation events while maintaining full-length tRNA coverage (Upton et al., 2021; K. Zhang et al., 2025). OTTR-seq uses a processive reverse transcriptase with a defined template-switching mechanism, improving the uniformity of read coverage across structured RNAs. This makes OTTR-seq particularly well-suited for transcriptome-wide profiling of tRNA modifications, where detecting both conserved and context-dependent modification events requires consistent capture of intact, full-length tRNAs. In archaeal species, where tRNAs are highly structured and often densely modified, this approach enables a comprehensive view of the modification landscape, revealing relationships between modification frequency, tRNA sequence, and enzyme specificity.

Much like individual aminoacyl-tRNA synthetases (aaRS) recognize and charge subsets of tRNAs with a specific amino acid (tRNA isoacceptors) based on distinct sequence and structural identity elements (Westhof et al., 2022), tRNA modification enzymes recognize specific sequence motifs and structural elements shared by a subset of tRNAs. Some modifications, such as N2-methylguanosine (m^2^G) or N2,N2-dimethylguansine (m^2^_2_G) at position 10, are important early in pre-tRNA maturation, mediating correct folding of the L-shape tertiary structure (Hori, 2019; Shigi et al., 2006). Transfer RNA modification pathways are interconnected and show cooperativity with one another, influencing stress response and disease through modulation of tRNA stability and decoding behavior (Hernandez-Alias et al., 2022; Sokołowski et al., 2018). To add to this complexity, the tRNA modification enzymes’ precise target specificity evolve across diverse phyla, causing uncertainty or inconsistencies between studies on the same family of modification enzymes in different model systems (de Crécy-Lagard et al., 2019).

Of the many tRNA modification types, methylation is the most common, with at least 13 different chemical variations of methylated RNA bases shared among all three domains of life (Hori, 2014; Jackman & Alfonzo, 2013; Motorin & Helm, 2011). Despite this overlap, modification enzymes vary widely in their protein domain architectures and substrate specificities across the tree of life. While some, like TrmI, function as multi-subunit complexes in eukaryotes but as a homotetramer in Archaea (Roovers et al., 2021), others show variability in domain architectures that influence tRNA recognition (Dixit et al., 2019). These differences impact how modification enzymes selectively recognize and act on diverse tRNA sequences, yet the extent of this variation and its functional consequences remain poorly understood. Investigating these evolutionary differences will provide insight into the determinants of tRNA modification specificity.

Here, we elucidate the landscape of RT-detectable tRNA modifications by applying OTTR-seq across nine archaeal species, including three thermophiles, two methanogens, two acidophiles, and two halophiles, to better understand the dynamic rate of tRNA modification evolution across different environments. In particular, we focus on position-specific modifications that vary between clades, isoacceptor-specific tRNAs, or a combination of the two. Furthermore, we investigate the distribution of corresponding tRNA modification enzyme orthologs and provide detailed co-evolutionary analyses of protein domain architectures with respect to variation in tRNA substrate target sites. Altogether, we find substantial evidence for conservation patterns for m^2^G/m^2^_2_G at position 6, m^1^A/m^1^G at position 9, m^2^_2_G/xG at position 10 and 26, which co-vary with the corresponding modification enzymes in these species, making prediction of the presence or absence of these modifications feasible from genome sequence alone.

## Results

### Detection of base-specific misincorporations reveals tRNA modification dynamics across archaea

**Figure 1.**
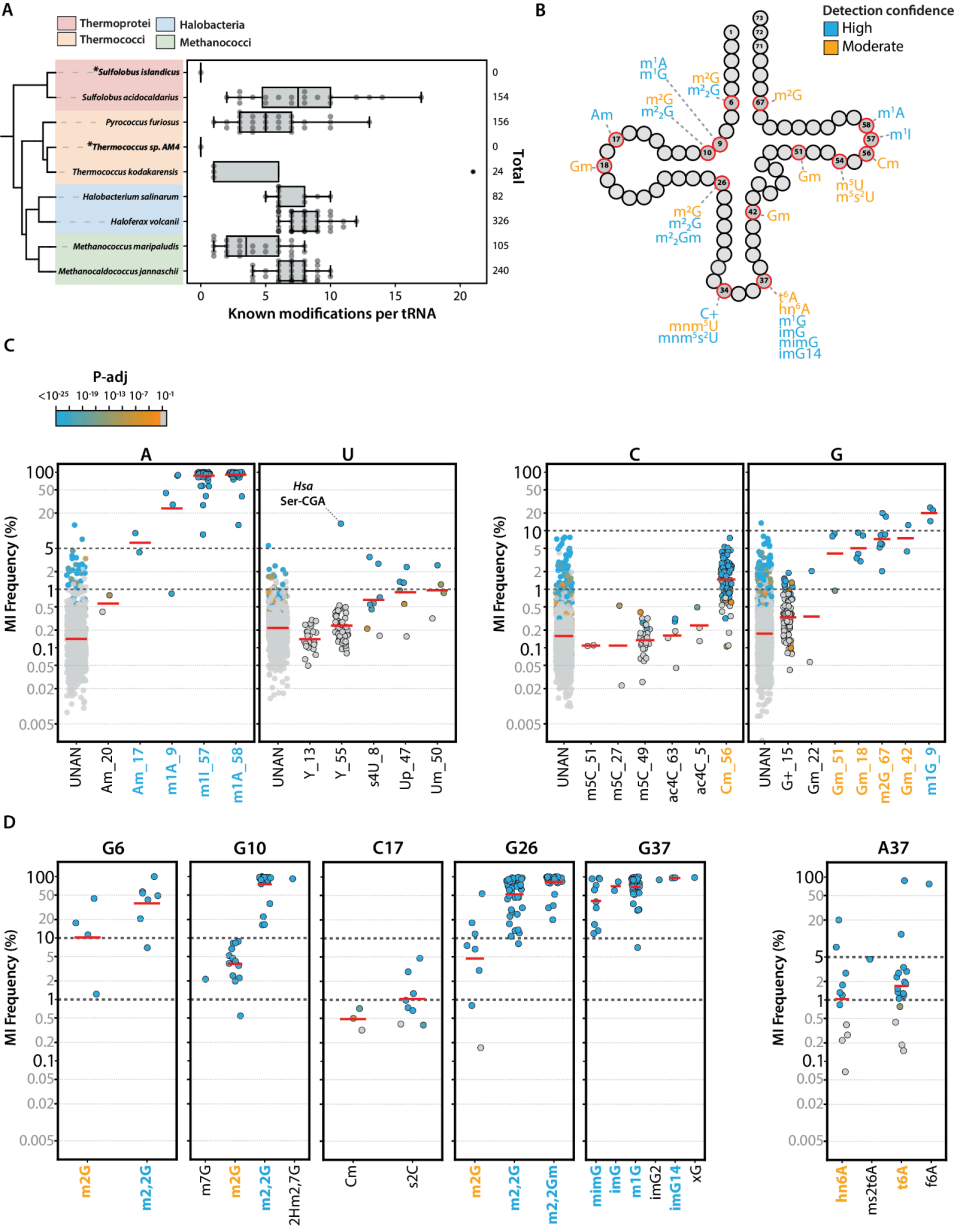
Known archaeal tRNA modifications and their misincorporations (MI) rates. A) Phylogenetic tree of representative archaeal species, annotated in boxplots, where each datapoint represents a given tRNA with annotated modifications. The number of known tRNA modifications per species was curated from Modomics and literature sources. Species wherein no literature sources define tRNA modifications are marked with an asterisk(*) B) Cloverleaf schematic of tRNA secondary structure indicating detection confidence level at positions of known tRNA modifications across all species. Modifications are color-coded by estimated confidence of detection via OTTR-seq, based on average misincorporation frequency (MI) and adjusted p-values. High confidence detection (blue) was classified by base-specific MI rates (mean MI > 10% for cytosine and guanine; mean MI >5% for adenine and uracil) and an adjusted p-value <0.05. Modifications with significant p-values but misincorporation rates below the respective base threshold were classified as moderate-confidence (orange). C) Position-specific MI frequencies stratified by base for unannotated and known modified bases. Each point represents a specific species tRNA. MI rates are plotted on a log scale where red lines indicate mean MI. Background MI for unannotated bases (UNAN) includes both unmodified and unannotated positions; edge lines on data points were omitted for clarity. Modifications are labeled below with associated position and modification type. Adjusted p-values represent the probability of observing a misincorporation frequency equal to or greater than the observed value (grey indicates p-adj > 0.05). D) MI profiles at selected positions where multiple known modification types can occur from the same base (e.g. G6, G10, C17, G26, G37, A37). Each subpanel shows MI distributions stratified by specific chemical modification. Differences in MI frequencies aid in distinguishing closely related modification types such as m^2^G vs. m^2^_2_G at G10. Confidence in detection varies across positions and modification types.

We profiled post-transcriptional tRNA modifications across nine archaeal species (Table 1) using OTTR-seq (Upton et al., 2021) to identify reverse transcription (RT)-induced misincorporation signatures of tRNA modifications (Figure 1). Known modification sites in six of these species (*H. volcanii, M. jannaschii*, *M. maripaludis*, *P. furiosus, S. acidocaldarius,* and *T. kodakarensis*) (Gupta, 1984; Hirata et al., 2019; Wolff et al., 2023; Yu et al., 2019) provided a validated reference for evaluating modification detection (Additional file: Table S0), while additional comparisons with *Thermococcus sp.* AM4 and *Sulfolobus islandicus* enabled assessment of short-term evolutionary conservation (Figure 1A). We predicted sites of tRNA modification via prominent and statistically significant misincorporation frequencies (MI frequency), defined as the proportion of sequencing reads at a given nucleotide position that differ from the reference base. High misincorporation frequencies define the extent to which a modification disrupts incorporation of the cognate nucleotide during reverse transcription, typically by disrupting Watson Crick (W-C) base pairing. The misincorporation frequency provides a proxy for the presence and stoichiometry of modification events.

To evaluate detection confidence, we implemented base-specific MI frequency thresholds that account for spontaneous misincorporation uniquely induced by each canonical base. Guanine and cytosine exhibited elevated background misincorporation rates, even in the absence of annotated modifications, which required adjusted criteria to reduce false positives. Sites of potential modification within tRNAs were classified as high-confidence if MI frequency exceeded 10% for G/C, while lower spontaneous MI frequencies allowed a tighter threshold of 5% for A/U. All high-confidence modification sites must also have statistical significance (adjusted p < 0.05). Positions with significant p-values but misincorporation rates below 10% for G/C or 5% of A/U were designated as moderate-confidence misincorporation sites. The distribution of high- and moderate-confidently detected modification is summarized by position (Figure 1B) and across all tRNAs (Additional file: Fig. S1 and Fig. S2, Table S1).

We observed that select tRNA modifications displayed a range of misincorporation behaviors. Modifications disrupting the W-C face (e.g., m^1^A, m^1^I, m^2^G, m^2^_2_G) consistently showed the highest MI rates (Figure 1C) and deviations from expected patterns revealed biologically meaningful variation. For example, while m^1^A58 typically results in near-complete misincorporation, Thermoprotei species showed reduced MI rates at A58 in tRNA^Ala^. Despite lower MI rates, the read identity (patterns of misincorporation) and read depth supported m^1^A modification at this position, suggesting substoichiometric modifications or tRNA-specific regulation (Figure 1C, Additional file: Fig. S3 and Fig. S4). Similar observations were made for A57 in *P. furiosus* Thr^GGU^_,_ where rates of misincorporation markedly dropped but the patterns of misincorporation were consistent with m^1^I.

Other notable outliers included pseudouridine at position 55, which was consistently undetectable across species, as expected given its minimal impact on reverse transcription. However, in *H. salinarum* Ser^CGA^, we observed an unusually high misincorporation rate and significant p-value at this position. While pseudouridine alone is unlikely to account for this signal, it may reflect the presence of a related or modified derivative such as m^1^Ψ, which has been reported at nearby positions like 54 but may occur at 55 in this specific context (Additional file: Fig. S4).

An important observation was the characteristic misincorporation behavior of different modification types occurring at the same position and base (Figure 1D, Additional file: Fig. S1). Modifications derived from guanine or adenine that disrupt the W-C face (e.g. m^1^A, m^2^G, and m^2^_2_G) tended to exhibit the highest misincorporation rates, making them more readily detectable. The relative distributions of these rates varied by modification type from the same position. In some cases, this allowed us to differentiate between modifications at the same position. For example, at position 10, the misincorporation frequency for m^2^_2_G was consistently higher than that for m^2^G, enabling distinction between these modifications.

### Homology-based predictions of tRNA modifications identifies dynamic hotspots

**Figure 2.**
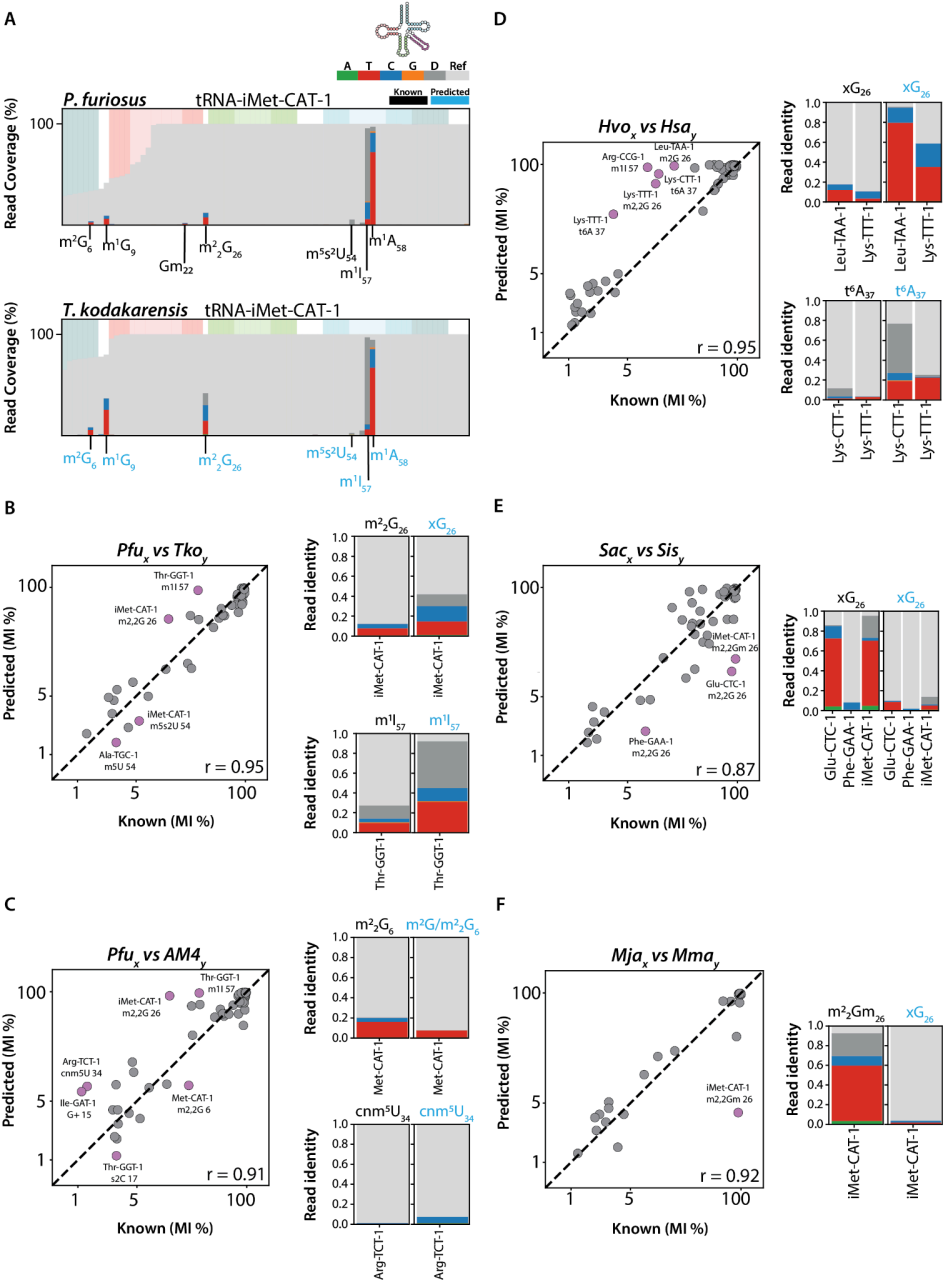
Overview of homology-based tRNA modifications predictions. A) Representative example of homology-based predictions for the initiator methionine (tRNA^iMet^) in *P. furiosus* (top) and *T. kodakarensis* (bottom). The coverage plot depicts the known modification sites indicated (e.g. black) and newly predicted sites highlighted (blue). Mapped-read coverage plot illustrates the read depth and pinpoint sites of modification. B,C,D,E,F) Pearson correlation between known modifications vs homology-predicted modifications. Each point in the scatter plot corresponds to a specific modification type and site from homologous tRNAs. The diagonal line represents perfect correlation (r=1) between known and predicted MI. Outliers (± 2 SD) are highlighted (purple) and annotated with their corresponding tRNA, modification, and position. The read identity (pattern of misincorporation) for select outliers are depicted next to the scatter plot, with known modification position (black) compared to newly predicted (blue) (See all outliers in Supplemental Figures) B) *P. furiosus (Pfu)* vs *T. kodakarensis (Tko)*. Select outliers iMet^CAU^ m^2^_2_G at position 26 and Thr^GGU^ m^1^I at position 57. C) *P. furiosus (Pfu)* vs *T. species* AM4 *(AM4).* Select outliers Met^CAU^ m^2^_2_G at position 6, and Arg^UCU^ cnm^5^U at position 34. D) *H. volcanii (Hvo)* vs *H. salinarum (Hsa)*. Select outliers Leu^UAA^ m^2^G, Lys^UUU^ m^2^_2_G at position 26, and Lys^CUU^, Lys^UUU^ t^6^A at position 37. E) *S. acidocaldarius (Sac) vs S. islandicus (Sis).* All outliers Glu^CUC^ and Phe^GAA^ m^2^_2_G, and iMet^CAU^ m^2^_2_Gm at position 26. F) *M. jannaschi (Mja)* vs *M. maripaludis (Mma).* Outlier iMet^CAU^ m^2^_2_Gm at position 26. For positions with more than one modification type (e.g., m^2^G/m^2^_2_G_26_) “x” was used. This notation is also used for most predictions, as exact modification types weren’t ascertained.

Mapping known modification sites to positions of misincorporations in previously characterized species allowed us to predict their presence in related species lacking experimental annotations. For example, the known modification profile of tRNA^iMet^ in *P. furiosus* was mapped to concordant sites of misincorporation in *T. kodakarensis*. In this case, we were able to predict all of the known and detectable modification sites in *T. kodakarensis*, with the exception of 2’-O-methyguanine (Gm) at position 22 (Figure 2A), which has a relatively low misincorporation frequency even in the *P. furiosus* tRNA (Figure 1C). Additional pairwise comparisons across species further supported the predictive power of misincorporation-based inference of site-specific tRNA modifications. Across comparisons, known and predicted misincorporation frequencies of modified homologous tRNAs were highly correlated (Additional file: Table S2). For species within the same clade, Pearson correlation coefficients ranged from 0.87 to 0.95 (Figure 2B–F, Additional file: Fig S5), and this strong agreement indicates that modified homologous tRNAs frequently share conserved misincorporation patterns, reinforcing the value of using known sites of tRNA modification as predictors for tRNA modification patterns in closely related taxa.

Outliers within these comparisons were particularly informative, revealing partial and variable modification states in homologous tRNAs between species within clades. As an example, (Figure 2B, Additional file: Fig. S6), the known m^2^_2_G in iMet^CAU^ of *P. furiosus* had a lower and more variable pattern of misincorporation compared to the predicted modification of the tRNA^iMet^ from *T. kodakarensis* at position 26. Interestingly, the predicted modification in *T. kodakarensis* had a larger number of deletions in the patterns of misincorporation. While the triple methylation of guanine (m^2^_2_Gm) is often associated with deletions and higher rates of misincorporations, we define all likely G-derived modifications at position 26 as “xG” as there are often two or more modification types that occur at this position that can not be consistently distinguished solely through MI frequencies. Similarly, the difference observed at position 57 in Thr^GGU^ shows that the patterns of misincorporation are consistent with m^1^I despite variation in rates of misincorporation. In *P. furiosus* vs *T. sp.* AM4 (Figure 2C), we identified additional outliers of known m^2^_2_G in iMet^CAU^ at position 6, known to have either m^2^G or m^2^_2_G. While *T. sp.* AM4 shows low rates of misincorporation that are often associated with m^2^G, we define all predicted G-derived modifications at this position as m^2^G/m^2^_2_G. The difference observed at position 34 in Arg^UCU^ shows that the pattern of misincorporation is consistent with cnm^5^U despite being at the lower limits of detection (~1% MI). Comparisons between *H. volcanii* and *H. salinarum* (Figure 2D) revealed several divergent misincorporation levels. The low rate and read identity of known m^2^_2_G in Lys^UUU^ in *H. volcanii* supports presence of m^2^G, which is consistent with the known m^2^G in Leu^UAA^. The predicted modifications in *H. salinarum* are consistent with the high rates of misincorporation often associated with m^2^_2_G. Due to the differences seen in these known modifications, we predict all G-derived modifications at position 26 as m^2^G/m^2^_2_G or xG. Additionally, modifications at position 37, such as t^6^A in Lys^CUU^ and Lys^UUU^ showed variations in the predictions, reflecting either partial modification or differences in species-specific regulation. In *S. acidocaldarius* vs *S. islandicus* (Figure 2E), position 26 modifications in Glu^CUC^, Phe^GAA^, and iMet^CAU^ stood out as underrepresented in *S. islandicus.* Despite these differences, the read identity patterns of predicted modifications were consistent with the respective known modifications. These variations may suggest species-specific differences in the regulation of these modifications. Finally, in *M. jannaschii* vs *M. maripaludis* (Figure 2F), position 26 in iMet^CAU^ was again identified as an outlier. In this case, the hyperthermophile (*M. jannaschii*) has identity patterns consistent with m^2^_2_Gm, whereas the mesophile (*M. maripaludis*) has patterns consistent with m^2^G.

Together, conserved modifications at positions like 6, 26, and 37 appear to be hotspots for interspecies variation in stoichiometry or type. These comparisons highlight how deviations from expected misincorporation frequencies can predict species-specific differences in modification state. Moreover, read identity patterns support the biological plausibility of these differences, as distinct patterns of misincorporation are often consistent with those of known modifications. Our observations emphasize the caution required in homology based modification predictions, where careful contextual interpretation is required. For our downstream analyses, we focus on modifications that have moderate to high confidence of detection, and use xN notation at positions where the exact modification type is unable to be inferred.

### Clade-specific patterns reveal coordinated modification in the acceptor stem and core regions

**Figure 3.**
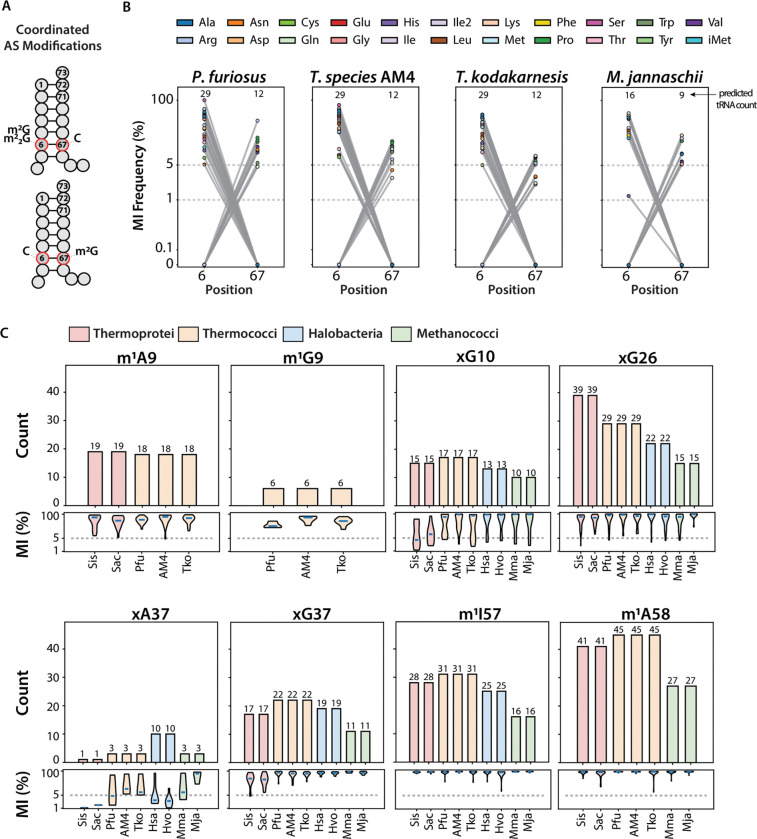
Clade-specific coordination patterns in the acceptor stem and conserved clade-specific tRNA modifications. A) Schematic representation of coordinated modification patterns at the acceptor stem of the tRNA cloverleaf secondary structure, highlighting a mutually exclusive relationship between position 6 (typically m^2^G or m^2^_2_G) and position 67 (m^2^G). Observed across multiple hyperthermophiles, the data suggest that these modifications do not co-occur within the same tRNA, possibly due to functional constraints. B) Paired MI frequencies for position 6 and 67 across four hyperthermophilic archaea: *P. furiosus, T. sp.* AM4*, T. kodakarensis,* and *M. jannaschii.* Each line connects paired MI values for individual tRNAs, with colored dots indicating isotype identity. Only moderate and high-confidence predictions (adjusted p-value < 0.05) are shown. Numbers above each panel indicates the total number of predicted modified tRNAs at positions 6 and 67, respectively. A pattern of coordinated modification is consistently observed. C) Distribution of MI frequencies for select moderate to high-confidence tRNA modifications shared across archaeal clades, grouped by phylogenetic class: Thermoprotei (salmon), Thermococci (tan), Halobacteria (blue), and Methanococci (green). Each violin plot summarizes the frequency and variation of a specific modification (e.g. m^1^A9, m^1^G9, m^2^G10, etc.) across species, with counts (above plots) indicating the number of modified tRNAs per group. This highlights the phylogenetic conservation and variability of tRNA modification patterns across lineages.

Distinct modification patterns in the acceptor stem and tRNA core regions reveal coordinated signatures that vary by clade. In particular, m^2^G and m^2^_2_G modifications at positions 6 and 67 (base pairing in the acceptor stem) are frequently predicted in hyperthermophilic species (Figure 3A). While prior studies have reported individual modifications at these positions in select tRNAs from *M. jannaschii* and *P. furiosus* (Wolff et al., 2023; Yu et al., 2019), the broader pattern of coordination has not been previously recognized. Our expanded analysis reveals that across multiple species, position 6 is more frequently modified than position 67, with misincorporation rates suggesting a predominance of m^2^_2_G6 over m^2^G6. In contrast, m^2^G67 is less frequent but consistently observed in distinct tRNA subsets (Additional file: Fig. S2 and Table S3). The two modifications are not predicted to co-occur within the same tRNA (Figure 3B), indicating a likely mutually exclusive pattern of modification. This trend is particularly evident in all Thermococci species and *M. jannaschii* (Methanococci). Most G6 conserved tRNAs are predicted to have a modification across these species (Additional file: Fig. S2 and Table S3). However, certain tRNAs in Thermococci, such as Glu^UUG^ and Ile^GAU^ , possess a C6, precluding the possibility of m^2^G or m^2^_2_G at this position in these cases.

Coordination of modification patterns is also observed between positions 10 and 26, two highly conserved sites of modification across archaeal tRNAs. While the predicted modifications conserved in species from Halobacteria and Methanococci tend to occur in mutually exclusive subsets of tRNAs, co-occurrence of modifications at both sites within the same tRNA is found in Thermococci and Thermoprotei (Additional file: Fig. S7 and Table S4). This pattern is especially pronounced in Thermoprotei, where multiple tRNAs such as tRNA^Asp^, tRNA^Glu^, and tRNA^Val^ are predicted to carry both m^2^G/m^2^_2_G10 and m^2^_2_G26 (Additional file: Fig. S2, S7 and Table S4). In contrast, while Thermococci also exhibits co-modification of position 10 and 26, their modification types differ. With lower rates of misincorporation at position 10, their tRNAs may instead carry m^2^G10 and m^2^_2_G26 (Additional file: Fig. S7). These differences are not entirely surprising as these specific clades span two phylums (Crenacrhaota and Euryarchaota). Their preferences in patterns of modifications suggest evolutionary divergence in enzymatic activity and substrate selection.

Within Euryarchaeota, coordinated patterns of G10 and G26 modification appear to be more closely linked to specific environmental variables such as temperature. Predicted G10 substrates typically contain G10oU25 identity elements, consistent with previous observations (Urbonavičius et al., 2006), while G26 modifications tend to occur in tRNAs lacking this pairing (Additional file: Fig. S8, Table S6). Interestingly, in Thermoprotei, several tRNAs predicted to be modified at both positions contain G10oU25 but lack the D-arm motifs (e.g., U13oU22, U13oA22) commonly associated with m^2^G10/m^2^_2_G10 in other clades. These findings suggest that the coordination of modifications at structurally proximal positions may reflect shared structural constraints in mesophilic species within Euryarchaeota, and the clade-specific tuning of enzyme-substrate interactions in thermoacidophiles within Crenarchota.

### Conserved and clade-specific tRNA modifications at functional positions support distinct evolutionary adaptations

While several tRNA modifications have been proposed to support adaptation to high-temperature environments, our results reveal additional patterns that suggest clade-specific evolutionary pressures beyond thermophily alone. A notable example is the methylation of purines at position 9, where we find clear distinctions in modification type and substrate specificity between Thermoprotei and Thermococci, which is consistent with previous work (Krishnamohan & Jackman, 2019). In Thermoprotei, predicted modifications are limited to m^1^A9, affecting most A9-containing tRNAs (Additional file: Fig. S2). By contrast, Thermococci species display a broader substrate range, with both m^1^A9 and m^1^G9 modifications predicted across a more diverse set of tRNAs (Additional file: Fig. S2). These differences coincide with distinct patterns of sequence conservation at position 9, such as the rare occurrence of C9 in specific tRNAs of Thermoprotei, and reflect lineage-specific recognition preferences of Trm10 homologs. Since species from both of these clades thrive in elevated temperatures, the variable targets for these tRNA modifications at conserved positions can be shaped by clade-specific substrate preferences and enzyme evolution, rather than by thermophilic adaptation alone.

**Figure 4.**
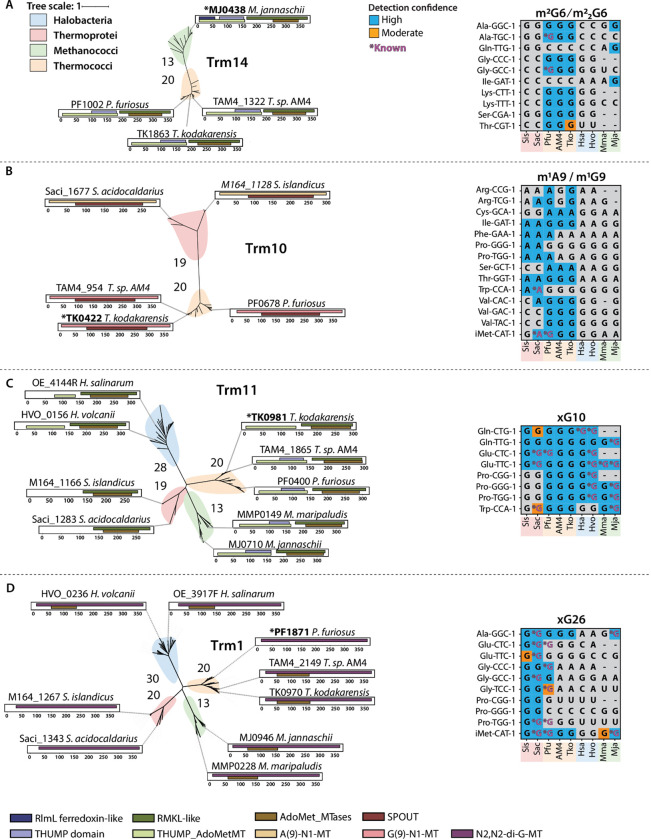
Phylogenetic and structural diversity of predicted tRNA modification enzymes and their target substrates across Archaea. Each panel presents a phylogenetic tree of predicted homologs of a specific tRNA-modifying enzyme (left), alongside a heatmap summarizing the confidence level of predicted substrates across species (right). Trees are constructed from clustered protein sequences colored by archaeal clades: Halobacteria (blue), Thermoprotei (salmon), Methanococci (green), and Thermococci (tan). Domain architecture annotations are shown on the tree branches, indicating key functional domains for three class I MTases (Trm14, Trm11, Trm1) and a class IV SPOUT MTase (Trm10). A) Trm14 homologs are restricted to Methanococci and Thermococci and are associated with predicted m^2^G6/m^2^_2_G6 modifications in specific tRNA substrates. The corresponding heatmap shows predicted modifications for representative tRNAs across genomes. B) Trm10 homologs are exclusive to Thermoprotei and Thermococci and are associated with m^1^A9 or m^1^G9 modifications. The heatmap displays inferred substrate preferences for these homologs across tRNAs and illustrates the dual-functional properties unique to Trm10 in Thermococci. C) Trm11 homologs are broadly conserved, although THUMP domain truncations are observed in Thermoprotei. These homologs are associated with m^2^_2_G10 modifications in select substrates. Divergence is most notable in tRNA^Pro^. D) Trm1 homologs show widespread conservation, with Thermoprotei variants displaying divergence in the AdoMet domain. Predicted modification patterns at position 26 include m^2^_2_G and other variants such as m^2^G/m^2^_2_Gm (xG26). Positions previously known and annotated as m^2^G reflect sites with adjusted p-values > 0.05, indicating lower statistical support. Together, these patterns highlight coordinated evolution of enzyme domain architectures and their target specificity across archaeal lineages.

Consistent with these patterns, we detected clear misincorporation signatures at position 9 in both clades, supporting the presence of m^1^A and m^1^G modifications in tRNA- and clade-specific manner (Figure 3C). Although Thermococci and Thermoprotei have a comparable number of clade-conserved m^1^A9-modified tRNAs predicted (n=18 and n=19, respectively), the individual tRNAs targeted differ between clades (Additional file: Fig. S2). For example, m^1^A9 is uniquely predicted in Pro^GGG^ in Thermoprotei, whereas in Thermococci m^1^A9 uniquely occurs in Cys^GCA^ and Ser^GCU^. G9-containing tRNAs are modified only in Thermococci (n=6), while Thermoprotei tRNAs with G9 remain unmodified. Additionally, Thermoprotei harbor rare C9-containing tRNAs, such as in Ser^GCU^ and tRNA^Val^, whereas Thermococci harbor A9/G9 in their respective tRNAs, further underscoring clade-specific variation in tRNA sequence contexts. Predicted modifications at position 9 appear restricted to these two thermophilic clades. No evidence for m^1^A9 and m^1^G9 was found in Halobacteria or Methanococci, which lack detectable Trm10 homologs (Figure 4A). Together, these patterns highlight a narrow conservation of position 9 modifications.

In addition to the clade-specific patterns observed at A9/G9, several other positions, such as G10 and G26, are modified across archaeal species, although their distribution and frequency vary by lineage and environmental niche (Figure 3C). Modifications at position 10, predicted as m^2^G or m^2^_2_G (xG), are present in all clades but are conserved with greater frequency between species in Thermococci (n=17) and Thermoprotei (n=15). Previous studies have associated m^2^_2_G10 with high-temperature growth in *T. kodakarensis* (Hirata et al., 2019; Hori, 2023), and our data suggest that the presence of m^2^_2_G10 does correspond with optimal growth temperatures, although it is not exclusive to thermophiles. For instance, these modifications are also frequent in mesophilic Halobacteria (n=13) and Methanococci species *M. maripaludis* (n=10). Interestingly, tRNA^Pro^ in Thermoprotei lacks a predicted modification at position 10 despite containing U25, a context known to promote m^2^_2_G10 installation for stabilization of the G10oU25 wobble pair (Urbonavičius et al., 2006). This absence suggests that the positioning and frequency of this modification may reflect lineage-specific adaptations that are not necessarily dependent on temperature alone.

Position 26 shows even border conservation, with nearly all species exhibiting high counts of predicted m^2^G/m^2^_2_G/m^2^_2_Gm (xG) at this site (Figure 3C). Thermoprotei have the largest number of predicted modifications at this position (n=39), followed by Thermococci (n=29), Halobacteria (n=22), and Methanococci (n=15). While m^2^_2_G and its hyper-modified variant m^2^_2_Gm have been associated with thermophilic species (Wolff et al., 2023), our results suggest that modification frequency at this position does not correlate strictly with optimal growth temperatures of these species. For example, acidophilic species exhibit high counts of predicted G26 modification, exceeding those observed in hyperthermophiles. Moreover, although the predicted number of conserved G26 is lowest in Methanococci, the hyperthermophile *M. jannaschii* independently shows broader modification patterns (n=21) (Additional file: Fig. S2). Importantly, across species, not all G26 tRNAs are predicted to be modified. These trends suggest that while G26 is conserved and frequently modified, the extent and specificity of its modification vary by clade, reflecting differences in tRNAs and the substrate recognition properties of Trm11 homologs.

Position 37, located adjacent to the anticodon, is another highly conserved site of modification, with distinct patterns of chemical diversity observed across archaeal clades (de Crecy-Lagard et al., 2010). Predicted modifications at this site fall into two main categories: xA37, which includes t^6^A-like derivatives, and xG37, which primarily includes m^1^G as well as wyosine-like derivatives. Our analysis shows that xA37 modifications are primarily restricted to Halobacteria, where they are predicted in 10 tRNAs conserved between the two species, with only a single conserved prediction detected in Thermoprotei (Figure 3C). This prediction corresponds to Ser^GCU^, an expected target across all archaeal species in this work (Additional file: Fig. S2). In addition, it is broadly expected in tRNAs that recognize codons beginning with adenine-ANN codons (W. Zhang & Westhof, 2025). However, we did not detect this highly conserved modification with confidence in Ser^GCU^ from *T. kodakarensis* or *P. furiosus*, likely due to the low misincorporation signal typically associated with t^6^A. In contrast, xG37 modifications are more broadly distributed and most prevalent in Thermococci, with up to 22 tRNA conserved between species, compared to lower counts in other clades (Figure 3C). These patterns highlight clade-specific distributions of distinct modifications at conserved sites and reflect both evolutionary divergence and technical limitations in detecting certain modification types.

Conserved modifications continue into the T-arm, particularly at position 57 and 58, sites known to contribute to tRNA structure and function across all domains of life (Anderson & Droogmans, 2005; Oerum et al., 2017; Swinehart & Jackman, 2015). Thermophilic and hyperthermophilic species frequently carry both m^1^I57 and m^1^A58 (Figure 3C), consistent with their proposed role in structural stabilization. In our analysis, m^1^I57 is among the most frequently predicted modifications, occuring in 48–65% of tRNAs per species (16/35 to 31/46). m^1^A58 is even more widespread, detected in 77–98% of tRNAs (27/35 to 45/46), with one notable exception: Halobacteria lack any predictions of m^1^A58 (n=0). This clade-specific absence contrasts with its near-universal presence in other archaeal groups, suggesting divergence of TrmI homologs which typically methylates both A57 and A58 (Roovers et al., 2021). These trends reinforce the high degree of conservation for modifications at structurally significant positions in the tRNA, while also revealing lineage-specific absence.

### Phylogenetic variation in tRNA methyltransferase architectures reveals enzyme specialization across Archaea

Methylation is one of the most common post-transcriptional modifications, found across all domains of life. The methyl donor used by most methyltransferases (MTases) is S-adenosyl-L-methionine (AdoMet), acting as their co-substrate. tRNA modification enzyme homolog distributions provide supporting evidence of methyltransferases having specialized roles that drive clade specific adaptations. The AdoMet-dependent enzymes are divided into five different classes based on their structural folds (I-V) (Bujnicki et al., 2002). The enzymes investigated in this work include three class I MTases (Trm14, Trm11, Trm1) and a class IV SPOUT MTase (Trm10). The phylogenetic relationships and predicted domain architectures of these enzymes across representative archaeal species suggest these enzymes have evolved independently towards different specificities (Figure 4). Each panel presents a phylogenetic tree depicting the presence, location, and range of predicted functional domains, alongside heatmaps summarizing the confidence of predicted substrates across species.

The THUMP-related Trm14 (m^2^G6/m^2^_2_G6) is distributed exclusively in hyperthermophiles, within the Methanococci and Thermococci clades. Consistent with known archaeal crystal structures (Fislage et al., 2012; Menezes et al., 2011), predicted Trm14 homologs maintain three conserved domains: an N-terminal ferredoxin-like domain (NFLD), a THUMP RNA-binding domain, and a Rossmann fold MTase domain (RFM) (Figure 4A, Additional file: Table S7). However, subtle variation at the N-terminal regions distinguishes homologs across clades, specifically MJ0438 uniquely contains a hit for a predicted RImL ferredoxin-like domain. Although there are variations in domain architecture among these homologs, differences in predicted substrate profiles indicate tRNA sequence divergence as the main factor influencing modification specificity rather than enzyme domain architecture alone. Interestingly, the enzyme responsible for m^2^G67 remains unidentified, with previous work suggesting Trm14 involvement (Hirata et al., 2019). Our homolog search revealed the presence of Trm14 paralogs distributed within these clades (Additional file: Table S11). These paralogs may represent alternative candidates responsible for m^2^G67.

Previously, the SPOUT domain modification enzyme Trm10 (m^1^G9/m^1^A9) was thought to be widely conserved across Archaea (Krishnamohan & Jackman, 2019), however with the availability of additional genomes, distribution of predicted Trm10 homologs is in fact quite narrow, unique only to species within Thermococci and Thermoprotei. Within these species, the characterization of TK0422 (TkTrm10) from *T. kodakarensis* has shown that it has remarkable dual specificity for N1-methylation of purine residues at position 9 (Krishnamohan & Jackman, 2019). Although SPOUT domains are notoriously challenging to model at the sequence level, InterProScan returned domain hits for features characteristic of a bifunctional tRNA (guanine(9)-/adenine(9)-N1)-methyltransferase from the NCBIfam domain database (Figure 4B, Additional file: Table S8). In contrast, Thermoprotei vary in their domain features that diverge from bifunctional characteristics and instead resemble residues of a tRNA (adenine(9)-N1)-methyltransferase. This interesting divergence between domain architectures suggests a functional split across phylogeny, and is further supported by the evidence of predicted target substrates from misincorporation (Figure 4B).

The broadly conserved THUMP-related modification enzyme Trm11 (m^2^G10/m^2^_2_G10) has distribution across all clades. Based on previous characterizations, TK0981 is expected to contain a NFLD, a THUMP domain, and a RFM, much like Trm14, differing only in the linker region connecting the NFLD and RFM domains (Hirata et al., 2016; Urbonavičius et al., 2006). Homologs in Thermoprotei have the conserved C-terminal AdoMet and RMKL-like domains, but the expected N-terminal THUMP domain is completely absent (Figure 4C; Additional file: Table S9). The absence of this important tRNA binding domain, along with predicted modified tRNA substrates, implies that the Trm11 in Thermoprotei has a modular partner that contributes to the binding of its targets. There is a predicted ortholog of the Trm112 activator protein present in both species, which has previously been shown to form a complex with Trm11 in the model *H. volcanii* (van Tran et al., 2018), but its contributions to binding tRNAs has not been characterized. The mode of binding by these divergent Trm11 homologs is not clear, however the predicted targeted substrates in species with divergent N-terminal regions do co-vary, specifically with tRNA^Pro^ (Figure 4C).

One major group of highly conserved tRNA methyltransferases is the Trm1 family (m^2^G/m^2^_2_G26). These MTases are unusual in that they do not possess a THUMP domain (Hori, 2023; Ihsanawati et al., 2008). Despite this, Trm1, similar to Trm11, catalyzes a two-step reaction, leading to mono- and dimethylation of guanosine. The protein comprises an N-terminal and C-terminal domain characteristic of class I MTases and uses AdoMet as a ligand for methylation. Predicted homolog domain architectures for this family broadly identifies the expected (guanine(26)-N(2))-dimethyltransferase (Additional file: Table S10) across all species, with AdoMet detected near the N-terminal (Figure 4D). This differs from Trm14 and Trm11, where AdoMet is typically found near the C-terminal. Surprisingly, InterProScan does not detect the AdoMet domain in all species, it diverges from expected residues used by the domain model (Additional file: Table S10). Despite relative similarity between the predicted domains across species, targeted substrates are drastically different (Figure 4D). In Euryarchaeota, there is a biased coordination between m^2^G10/m^2^_2_G10 and m^2^_2_G26 (Additional file: Fig. S7), which could imply one is the anti-determinant of the other. In Thermoprotei, both G10 and G26 are predicted to be modified with m^2^_2_G.

### tRNA sequence and structure features reveal determinants and anti-determinants of modification enzyme targeting

**Figure 5.**
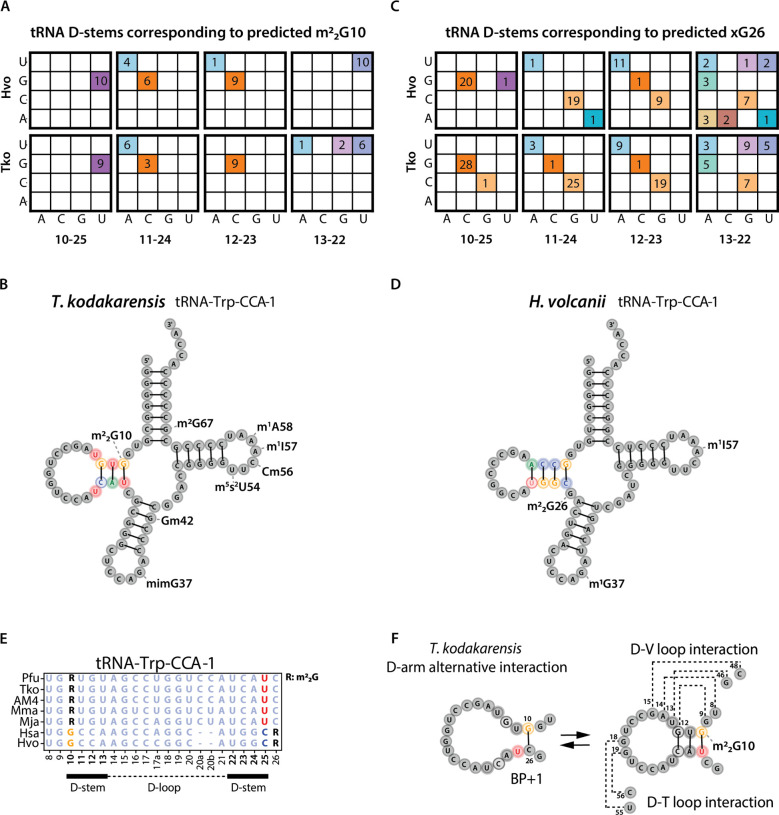
Conserved residues show evidence for a bias in recognition elements of predicted m^2^_2_G10 and xG26 modified tRNAs. A) Determinants corresponding to high confidence predictions of m^2^_2_G10 in *T. kodakarensis* (Tko) and *H. volcanii* (Hvo). Colors represent base pair identities. B) Determinants corresponding to high confidence predictions of and m^2^_2_G26, similar to panel A. C) Predicted secondary structures of tRNA^Trp^ from *T. kodakarensis* with highlighted Trm11 identity elements. D) tRNA^Trp^ from *H. volcanii* with highlighted Trm11 identity elements. E) Alignment of tRNA^Trp^ with previously characterized identity elements for Trm11 colored by nucleotide convention. Predicted modifications across species corresponding to identity elements. F) Conceptual illustration of weak D-arms depending on m^2^_2_G10 to prevent alternative interaction.

Positions with predicted modifications correspond to conserved nucleotide features in specific tRNAs across various species. Likewise, shifts in predicted modifications are linked to changes in nucleotides at distinct positions. Our comprehensive analysis across multiple archeal clades expands upon previous observations, reinforcing and broadening the understanding of identity elements critical for modification enzyme recognition. Specifically, the strongest association of these nucleotides aligns with previously characterized identity elements for Trm11, requiring G10oU25 pair in the D-arm (Urbonavičius et al., 2006). Our broad sampling across archaeal species supports this identity element, reinforcing its conserved functional importance. Among Euryarchaeota species, the frequency of predicted m²G10/m²_2_G10 modifications correspond with the expected G10oU25 and U13oU22 (Figure 5A). Moreover, our analysis identifies additional correlations, involving other non-Watson-Crick pairs such as U13oG22 and U13oUA22 in Thermococci and Thermoprotei (Additional file: Fig. S8, Table S5).

Non-Watson-Crick paris, particularly GoU paris, are known to induce structural variations essential for protein binding without necessitating direct protein-base contact (Westhof et al., 2020, 2022). Our extensive cross-clade analysis further supports that these structural variations contribute to enzyme recognition. Structurally, residues forming Non-Watson-Crick pairs result in shorter, less stable D-stems, which often correspond with the presence of predicted m²_2_G10 modifications (Figure 5B & E). Conversely, we observed absences of these modifications in tRNAs possessing more stable D-stems, further supporting the functional role that this modification has in the structural integrity of tRNAs. For instance, within Halobacteria, the tRNA^Trp^ deviates from the conserved G10oU25 pair, instead forming a G10oC25 pair, coinciding with the absence of predicted m²_2_G10 modification (Figure 5C–D). This difference highlights the stringent substrate specificity linked to identity elements like U25, essential for facilitating m²_2_G10-U25 interactions, but not m²_2_G10-C25 interactions.

Similarly, nucleotide features specific to predicted targets of G10oU25 containing tRNAs have been identified as anti-determinants for the action of Trm1, as demonstrated previously in *P. furiosus* (Urbonavičius et al., 2006). Our results support this relationship across Euryarchaeota, showing a strong association between the presence of G10oU25 and the absence of m²_2_G26 (Figure 5E). Interestingly, Thermoprotei do not exhibit this exclusion pattern and often show co-modification of m²_2_G10 and m²_2_G26, in addition to unique targeting of C12=G23 and A12oU23 tRNAs (Additional file: Fig. S8, Table S6). This divergence highlights structural adaptation strategies that may be unique to Thermoprotei. In general, weak D-arms likely depend on m²_2_G10 to stabilize the tRNA in its functional L-shaped conformation (Steinberg & Cedergren, 1995). In a specific example of the tRNA^Trp^ of *T. kodakarensis*, we see how m²_2_G10 may prevent an alternative interaction between G10=C26 and likely plays a role in most m²_2_G10oU25-containing tRNAs (Figure 5F). Collectively, this multi-clade analysis significantly extends our current knowledge on interplay between tRNA sequence, structure, and modification enzymes.

## Discussion

Through interrogating high-throughput sequencing datasets alongside genomic analyses we have expanded the scope of our understanding of post-transcriptional tRNA modifications, uncovering novel relationships, evolutionary implications, and insights into the coevolution of tRNA substrates with their modifying enzymes. Our comprehensive analysis across diverse archaeal species reveals distinct modification patterns reflecting adaptive mechanisms shaped by both environmental pressures and phylogenetic constraints.

Our base-specific misincorporation analysis illustrates how modifications differentially impact reverse transcription behavior, influencing their detectability via RT-based methods. Modifications disrupting W-C base pairing, such as m^2^_2_G and m^1^A, consistently yield higher misincorporation signals, facilitating robust detection. Conversely, modifications like 2’-*O*-methylations (Am, Cm, Gm, Um) remain challenging to detect due to minimal interference with reverse transcription or shadow signals resulting from proximity to nearby modifications. These are important examples of methodological limitations that warrant caution when inferring presence or absence solely from low misincorporation signals, particularly at known conserved modification sites such as position 56.

In this study, we predicted xG modifications at several positions, finding surprising relationships, such as the coordination of m^2^G/m^2^_2_G at positions 6 and 67 in the acceptor stem of hyperthermophiles. While previous work noted individual modifications at these positions, our comparative approach clearly indicates their mutual exclusivity, suggesting a previously unrecognized functional interdependence. Despite narrow conservation in hyperthermophiles, these modifications may not play a role in thermal-stability, which typically involves T-loop/D-loop interactions at the outside globular corner of the L-shape (Grosjean & Oshima, 2007). Insights from (Wolff et al., 2023) suggest m^2^_2_G6-C67 disrupts the W-C base pair, resulting in a pronounced propeller twist, likely playing a role in modulating the aminoacylation efficiency of aaRS and tRNA charging. With our new observations of this coordinated relationship, we hypothesize that as the steric disruptions alter between m^2^_2_G6-C67 and C6-m^2^G67, charging efficiencies may be regulated for specific tRNAs. It’s tempting to speculate that this unique mechanism of charging regulation could provide these hyperthermophiles with adaptive strategies to alter protein synthesis rates in response to environmental shifts.

Additionally, we identified coordinated modification patterns involving conserved core positions 10 and 26 across multiple archaeal clades. Our results highlight distinct clade-specific modification preferences, particularly in Thermoprotei, where co-occurrence of m^2^_2_G10 with m^2^_2_G26 is surprisingly frequent. While Thermococci share the unique co-modification of G10 and G26, their patterns contrast with lower frequencies of misincorporation that suggest pairing of m^2^G10 with m^2^_2_G26 is predominant. This difference implies evolutionary divergence in enzyme specificity for substrate recognition strategies, possibly linked to adaptations beyond thermal stability. Prior biochemical work supports G10oU25 pairs as a robust identity element of m^2^_2_G10 by Trm11, while simultaneously acting as an anti-determinant for Trm1-mediated m^2^_2_G26 modifications (Urbonavičius et al., 2006). Interestingly, our observations in Thermoprotei challenge this established pattern, revealing frequent coexistence of both modifications despite expected anti-determinant, indicating flexibility in recognition mechanisms or alternative stabilization strategies. Structurally, these species may utilize the unusual co-modification of 10 and 26 to fold the D-arm region and stabilize the three-dimensional core structure. As a general rule, methylations promote precise H-bonded pairs (e.g. m^1^A favors Hoogsteen pairs, m^2^G/m^2^_2_G favors GoU pairs) and electrostatic charges introduced by the chemical adducts are localized in shielded pockets of the tRNA fold (m^1^A, m^7^G, G^+^). In addition, methylations on the base and/or the ribose affect the hydration shells in complex ways (Auffinger & Westhof, 2001; Wolff et al., 2023). These intricate interactions between position 10 and 26 may reflect nuanced structural and functional roles, suggesting novel adaptations to otherwise unstable D-arm configurations, and ensuring tRNA stability and proper folding in acidic environments with elevated temperatures.

The broad identification of tRNA modification enzyme homolog distributions provides supporting evidence for methyltransferases having specialized roles that drive clade-specific adaptations. Comparative analyses of enzyme domain architectures clearly indicate independent evolutionary trajectories towards distinct chemistries and substrate specificities. A distinct example of this is the divergence of Trm10, where Thermoccoci possess dual-specific m^1^A9/m^1^G9 MTase functionality, contrasting Thermoprotei’s exclusive m^1^A9 specificity. Another example is seen in Trm11, where predicted domain architectures indicate a complete loss of the THUMP domain, specifically within Thermoprotei. The absence of this critical domain, along with differences in predicted modified tRNA substrates, suggests a potential modular partnership with another protein to facilitate substrate binding. In support of this speculation, both Thermoprotei species analyzed here possess predicted orthologs of the Trm112 activator protein, known from studies in *H. volcanii* to form complexes with Trm11(van Tran et al., 2018). Together, the divergence of Trm11 domain architectures, combined with unique targeting of C12=G23 and A12oU23 tRNAs in Thermoprotei, are illustrative examples of enzyme and substrate coevolution. Additional comparative insights from recent biochemical analysis of the Trm1 homolog in humans (TRMT1) further supports nuance specificities of enzyme-substrate recognition. Human TRMT1 independently catalyzes m^2^G or m^2^_2_G in a substrate-dependent manner, governed by specific base pairs in the D-stem (Xiong et al., 2023). These similar structural codes may apply broadly across domains, potentially reflecting deep evolutionary conservation of modification determinants.

Our broad analysis provides evidence reinforcing and expanding upon previously identified tRNA modification determinants and anti-determinants, as well as revealing cases where these established patterns are challenged, particularly in Thermoprotei. However, other modification sites, such as those predicted to carry m²G6/m²_2_G6, m^1^A9/m^1^G9, and m²G67, do not exhibit strong evidence for associations with specific nucleotide residues within the tRNA body. Previous biochemical studies on Trm10 have suggested that substrate preferences may instead be influenced by differences in the overall stability of the folded tRNA structure. It is possible that for enzymes such as Trm10 and Trm14, substrate recognition relies on interactions with the global L-shaped tertiary structure in combination with precise recognition of the catalytic site nucleotide (Strassler et al., 2022). This distinction demonstrates the diversity of recognition strategies across modification enzymes, and emphasizes that inferring determinants of modifications for many enzymes likely requires integration of tertiary structural context.

Altogether, this work generated a wealth of high-throughput data that lays the groundwork for further research by contributing detailed information on tRNA modification profiles, substrate preferences, enzyme distributions, and evolutionary patterns across archaeal lineages. We present a comprehensive experimental analysis of archaeal tRNA modifications and associated enzymes, enhancing our understanding of their adaptive roles. These results provide a valuable foundation for guiding targeted biochemical validation experiments, structural studies of enzyme-substrate interactions, and development of mechanistic models that explain modification specificity and coordination. Future research should focus on characterizing the mechanisms responsible for these modifications, exploring their regulatory functions in translation, and leveraging archaeal strategies for functional applications.

## Conclusions

This study provides a comprehensive experimental analysis of archaeal tRNA modifications and the enzymes predicted to install them. By combining high-throughput sequencing with comparative genomic analysis, we uncovered coordinated and clade-specific modification patterns at key tRNA positions (i.e. 6, 9, 10, and 26) and linked these patterns to evolutionary changes in enzyme specificity and domain architecture. Our findings extend the known repertoire of identity elements and anti-determinants of Trm11 and Trm1 homologs, while also revealing instances where recognition rules are challenged, particularly within Thermoprotei. These results emphasize co-evolution between tRNA structure, modifications, and their enzymes in the context of extreme environmental adaptation. Beyond mapping, this work establishes a framework for future biochemical validation, predictive modeling, and functional studies of tRNA modifications. Ultimately, the insights gained here advance our understanding of tRNA biology in Archaea and provide new opportunities for leveraging tRNA modifications for functional applications.

## Methods

### Culture and RNA isolation

Cultures of *M. maripaludis*, *M. jannaschii*, *S. islandicus*, *S. acidocaldarius*, *T. kodakarensis, T. sp.* AM4, *P. furiosus*, *H. salinarum* and *H. volcanii* were prepared as described previously (Crowley et al., 2006; Jäger et al., 2014; Liman et al., 2022; Mukhopadhyay et al., 1999; Tang et al., 2009; Wagner et al., 2012; Whitman et al., 1987; C. Zhang et al., 2013). For *T. kodakarensis* and *H. volcanii,* we had three biological replicates available (Additional file: Fig. S9). Total RNA from archaeal cell pellets were extracted as described previously in (Tsai et al., 2025). Briefly, cells were treated with BAN reagent (Molecular Research Center, Inc., Cat #BN191). RNA was purified with equal volume of acid phenol-chloroform with isoamyl alcohol (125:24:1, Thermo Fisher Scientific, Cat #AM9722) was added to the reaction and centrifuged to separate the aqueous phase from the organic phase. RNA was then precipitated with 1.5 volumes of isopropanol and 0.1 volume of sodium acetate (Sigma Aldrich, Cat #S7899) at −20C overnight. Finally, the precipitated RNA pellets were washed with 75% ethanol and dissolved in nuclease-free water. Total RNA was used as input for library construction.

### OTTR-seq library construction

Total RNA was used for generating OTTR-seq libraries, as previously described (Upton et al., 2021). Slight adjustments to the protocol were made to improve tRNA capture. Briefly, total PNK-treated RNA was 3’ tailed using mutant BoMoC RT in buffer containing only ddATP for 90 minutes at 30°C, with the addition of ddGTP for another 30 minutes at 30°C. This was then heat-inactivated at 65°C for 5 minutes, and unincorporated ddATP/ddGTP were hydrolyzed by incubation in 5 mM MgCl2 and 0.5 units of shrimp alkaline phosphatase (rSAP) at 37°C for 15 minutes. 5 mM EGTA was added and incubated for 100°C for 5 minutes to stop the reaction and denature GC-rich tRNAs. Reverse transcription was then performed at 37°C for 30 minutes, followed by heat inactivation at 70°C for 5 minutes. The remaining RNA and RNA/DNA hybrids were then degraded using 1 unit of RNase A at 50°C for 10 minutes. cDNA was then cleaned up using a MinElute Reaction CleanUp Kit (Qiagen). To reduce adaptor dimers, cDNA was run on a 9% UREA page gel, and the size range of interest was cut out and eluted into gel extraction buffer (300mM NaCl, 10mM Tris; pH 8.0, 1mM EDTA, 0.25% SDS) and concentrated using EtOH precipitation. Size-selected cDNA was then PCR amplified for 12 cycles using Q5 High-fidelity polymerase (NEB #M0491S) and buffer pack with high GC Enhancer (NEB #B9027S). Amplified libraries were then run on a 6% TBE gel, and the size range of interest was extracted to reduce adaptor dimers further. Gel slices were eluted into a gel extraction buffer (300mM NaCl, 10mM Tris; pH 8.0, 1mM EDTA) followed by concentration using EtOH precipitation. Final libraries were pooled and sequenced using 150 SE and 200 cycle kits on an Illumina NovaSeq.

### tRNA Analysis of eXpression (tRAX)

Sequencing adaptors were trimmed from raw reads using cutadapt, v1.18, and read counts were generated for RNA types tRAX (Holmes et al., 2022). Briefly, trimmed reads were mapped to their respective strain genomes: *Methanococcus maripaludis* S2, *Methanocaldococcus jannaschii* DSM 2661, *Sulfolobus islandicus* M.16.4, *Sulfolobus acidocaldarius* DSM 639, *Thermococcus kodakarensis* KOD1, *Thermococcus* sp. AM4, *Pyrococcus furiosus* DSM 3638, *Halobacterium salinarum* R1, and *Haloferax volcanii* DS2. Each of the references were combined with their mature tRNAs obtained from GtRNAdb (Chan & Lowe, 2016). Trimmed reads were mapped using Bowtie2 in very-sensitive mode with the following parameters to allow for a maximum of 100 alignments per read: –very-sensitive –ignore-quals –np 5 -k 100. Mapped reads were then filtered to retain only the “best mapping” alignments. Raw read counts of tRNAs and other small RNA types were computed using tRNA annotations from GtRNAdb, and annotations from Refseq. Raw read counts were then normalized using DESeq2.

### tRNA modification analysis

Sites of known tRNA modifications were compiled from Modomics (Cappannini et al., 2023) and relevant literature (Hirata et al., 2019; Krishnamohan et al., 2019; Wolff et al., 2023; Yu et al., 2019). Modification types for *M. maripaludis*, *M. jannaschii*, *S. acidocaldarius*, *T. kodakarensis, P. furiosus*, and *H. volcanii* were curated in a tab delimited table (Supplementary table) and mapped to their corresponding tRNA sequence alignments. Known modification positions were then mapped onto orthologous tRNAs in unannotated species within phylogenetic clades, focusing on sites with conserved reference nucleotides and shared rates of misincorporation.

To assess whether observed misincorporation frequencies were statistically significant relative to background, we applied tMAP (https://github.com/Aimann/tMAP)(K. Zhang et al., 2025) using aligned tRNA reads from tRAX (Holmes et al., 2022) as input. At each position misincorporation frequency was calculated as the number of non-reference base calls divided by total read coverage. A beta distribution was modeled using these empirical counts, incorporating pseudocounts derived from the mean and variance of misincorporation rates at known modified positions. A modification threshold was empirically determined as the 75th percentile of misincorporation rates, stratified by reference base identity. P-values were computed using the cumulative distribution function of the beta distribution, representing the probability of observing a misincorporation frequency equal to or greater than the observed value under the null model.

Modification detection relied on two key metrics: the observed misincorporation rate at each position, and the statistical significance (adjusted p-values) of these observed rates. We implemented a base-specific thresholding strategy that accounts for background misincorporation rates inherent to each base. We observed that cytosine and guanine-derived positions tend to exhibit elevated background rates, even at unannotated sites, exceeding 5% and resulting in statistically significant p-values despite the likely absence of true modification. To reduce risk of false-positive predictions driven by these elevated baselines, we set higher thresholds for levels of confidence in these contexts. Specifically, a position and modification type was classified as high-confidence if it met a base-specific misincorporation (>10% for cytosine and guanine; >5% for adenine and uracil) and an adjusted p-value <0.05. Modifications with significant p-values but misincorporation rates below the respective base-specific threshold were classified as moderate-confidence. This approach preserves sensitivity for modifications with potential to induce RT misincorporation, while improving specificity in contexts where signals vary.

Across species, modification types, and positions we observed that variation in detection was largely attributable to differences in the distribution of modification rates across sequence contexts and tRNA positions. The p-value reflects how a given misincorporation rate is relative to the expected background for a specific base. Even if two different sites have the same rate, they may differ in statistical significance if one falls within a narrow, low-background distribution and the other does not. While low coverage occasionally limited detection, the primary factor influencing statistical significance was the depth and magnitude of misincorporation at a given site.

In cases where multiple modification types are known to occur at the same position, relative misincorporation frequency was used to infer the likely modification. For example, m^2^_2_G generally causes higher misincorporation than m^2^G at position 10, enabling us to discriminate between these two forms in specific archaeal species. This distinction was also partially observable at positions 6 and 26, though with reduced confidence. In some instances, misincorporation patterns were not sufficiently distinct to unambiguously infer modification type. In such cases, known modification types from closely related species with identical reference bases were used to assign a most-likely modification classification.

### Homolog search and domain analysis

To search for archaeal homologs and compare domain architectures, we collected representative tRNA modification enzymes that were previously characterized (Constantinesco et al., 1998; Jackman et al., 2003; Menezes et al., 2011; Urbonavičius et al., 2006). Each representative was queried across 220 archaeal genomes using BLASTp with default parameters, then results were filtered to select hits with the lowest e-value for each species and representative. Domain analysis and annotation of highest scoring hits was performed using the InterProScan software package (v5.65–97.0) with the FunFam (4.3.0), MobiDBLite (v2.0), NCBIfam (v13.0), PANTHER (v18.0), SuperFamily (v1.75), CDD (v3.20), Pfam (v36.0), SMART (v9.0), PRINTS (v42.0) and PIRSF (v2023_05) databases. As e-values are specific to each InterPro database and each utilizes their own e-value post-processing, Pfam and CDD were used preferably when domains were found. All matches were considered tentative hits (https://interproscan-docs.readthedocs.io/en/latest/FAQ.html).

### Phylogenetic analysis

The evolutionary relationships among each of the tRNA modification enzymes were inferred using maximum likelihood phylogenies derived from clustered BlastP hits of representatives and their subsequent multiple sequence alignments. In brief, hits for each representative were clustered using MMseq2 (Steinegger & Söding, 2017) using the parameters --min-seq-id 0.3 --cov-mode 0. Clustered groups were subset and aligned with MAFFT using default parameters, then concatenated and aligned again using the parameters --retree 1 --maxiterate 0.

All maximum likelihood phylogenetic trees were estimated using IQ-TREE 2 (v2.1.0). Selection of the best-fit model of amino acid substitution was inferred for the phylogenies using the ModelFinder function in IQ-TREE (LG+F+R6). Custom python scripts were used to prune trees by keeping only BlastP hits of each representative with the lowest e-value in a genome. Trees of each representative and its best-scoring homologs were plotted using the Interactive Tree Of Life (https://itol.embl.de). Predicted domain architectures were annotated onto the phylogenies in Adobe Illustrator.

### tRNA secondary structure

The R2DT software (McCann et al., 2025) was used to predict and illustrate tRNA secondary structures. Adobe illustrator was used to annotate structures with predicted modifications and tertiary interactions.

## Supplementary Material

Supplement 1

## Figures and Tables

**Table 1.** Summary of species studied in this paper.

Species	Phylum	Class	Habitat	Temperature Preference	Number of tRNA Genes
*Sulfolobus islandicus*	Crenarchaeota	Thermoprotei	Volcanic hot springs	Thermoacidophile (75°C)	45
*Sulfolobus acidocaldarius*	Crenarchaeota	Thermoprotei	Terrestrial hot springs	Thermoacidophile (75°C)	46
*Pyrococcus furiosus*	Euryarchaeota	Thermococci	Hydrothermal marine sediments	Hyperthermophile (95°C)	46
*Thermococcus sp.* AM4	Euryarchaeota	Thermococci	Sea hydrothermal vents	Hyperthermophile (80°C)	46
*Thermococcus kodakarensis*	Euryarchaeota	Thermococci	Sea hydrothermal vents	Hyperthermophile (85°C)	46
*Haloferax salinarum*	Euryarchaeota	Halobacteria	Hypersaline lakes and salterns	Extreme halophile (40°C)	47
*Halobacterium volcanii*	Euryarchaeota	Halobacteria	Hypersaline environments	Moderate halophile (42°C)	47
*Methanococcus maripaludis*	Euryarchaeota	Methanococci	Salt marshes	Mesophile (37°C)	35
*Methanocaldococcus jannaschii*	Euryarchaeota	Methanococci	Marine hydrothermal vent	Hyperthermophile (85°C)	34
